# The Role of Polyphosphate in Motility, Adhesion, and Biofilm Formation in *Sulfolobales*

**DOI:** 10.3390/microorganisms9010193

**Published:** 2021-01-18

**Authors:** Alejandra Recalde, Marleen van Wolferen, Shamphavi Sivabalasarma, Sonja-Verena Albers, Claudio A. Navarro, Carlos A. Jerez

**Affiliations:** 1 Laboratory of Molecular Microbiology and Biotechnology, Department of Biology, Faculty of Sciences, University of Chile, Santiago 8320000, Chile; alejandra.recalde@biologie.uni-freiburg.de (A.R.); clnavarrol@gmail.com (C.A.N.); 2 Laboratory of Molecular Biology of Archaea, Institute of Biology II-Microbiology, University of Freiburg, 79085 Freiburg, Germany; marleen.van.wolferen@biologie.uni-freiburg.de (M.v.W.); shamphavi.sivabalasarma@biologie.uni-freiburg.de (S.S.); sonja.albers@biologie.uni-freiburg.de (S.-V.A.)

**Keywords:** sulfolobales, Saccharolobus solfataricus, Sulfolobus acidocaldarius, inorganic polyphosphates, biofilm, archaellum

## Abstract

Polyphosphates (polyP) are polymers of orthophosphate residues linked by high-energy phosphoanhydride bonds that are important in all domains of life and function in many different processes, including biofilm development. To study the effect of polyP in archaeal biofilm formation, our previously described *Sa. solfataricus* polyP (−) strain and a new polyP (−) *S. acidocaldarius* strain generated in this report were used. These two strains lack the polymer due to the overexpression of their respective exopolyphosphatase gene (*ppx*). Both strains showed a reduction in biofilm formation, decreased motility on semi-solid plates and a diminished adherence to glass surfaces as seen by DAPI (4′,6-diamidino-2-phenylindole) staining using fluorescence microscopy. Even though *arl*B (encoding the archaellum subunit) was highly upregulated in *S. acidocardarius* polyP (−), no archaellated cells were observed. These results suggest that polyP might be involved in the regulation of the expression of archaellum components and their assembly, possibly by affecting energy availability, phosphorylation or other phenomena. This is the first evidence indicating polyP affects biofilm formation and other related processes in archaea.

## 1. Introduction

Polyphosphates (polyP) are polymers of inorganic orthophosphate that can reach hundreds to thousands of residues in size. PPK and PPX are the two main enzymes related to polyP metabolism in Prokaryotes. PPK synthesizes polyP, consuming ATP as the polyP chain grows, generating ADP and polyP (n+1) as final products. On the other hand, PPX degrades polyP starting from its last phosphate group, releasing Pi and polyP (n−1) in the process [1]. In archaea, PPX has been identified either by experimental [2] or bioinformatic analysis [3]. Nevertheless, even though polyP accumulates in some Crenarchaeotes, no archaeal PPK homolog has been found so far, suggesting a different enzyme involved in polyP synthesis in this phylum.

Many functions of polyP have been revealed in bacteria such as: ATP substitution, helping in nutrition starvation, functioning in metal resistance, and several other roles [1,4]. Some of those functions are related to biofilm formation, motility, and sporulation [5,6,7,8] as shown with several PPK and/or PPX mutants in diverse bacterial species.

A *ppk* deletion mutant in *Pseudomonas aeruginosa* showed a thinner and more uniform biofilm when compared to the wild type (WT) strain and lacked cells forming clusters along with water channels [6]. This mutant was defective in swimming, swarming, twitching and adhesion to surfaces. All of these phenomena are related to biofilm formation. Involvement of polyP in biofilm development, sporulation and even virulence was also shown in *Bacillus cereus* [7] and *Campylobacter jejuni* [5].

In *E. coli*, the role of polyP in biofilm generation has been studied in more detail. Rather than the intracellular level of the polymer, polyP degradation triggers biofilm formation in stationary phase via Type 2 Autoinducers (AI-2), specifically the LuxS Quorum Sensing system [9]. A correlation between polyP levels and AI-1 and AI-2 was also seen in *P. aeruginosa* [6].

The possible role of polyP in biofilm formation in archaea has not been explored. Archaea lack AI-2, suggesting that regulation in these microorganisms functions differently. Regulation of biofilm formation in *Sulfolobus acidocaldarius* involves two transcriptional regulators belonging to the Lrs-14 family: encoded by Saci_1223 and Saci_0446, the latter is known as AbfR1 (Archaeal Biofilm Regulator 1) [10]. Saci_1223 is a positive regulator, inducing biofilm formation, whereas AbfR1 is a negative regulator that inhibits biofilm development by repressing extracellular polymeric substances (EPS) synthesis and promoting swimming motility by using archaella. AbfR1 is regulated by phosphorylation [11] and cannot impair biofilm formation when phosphorylated. The pathway for EPS synthesis has not been described yet for *Sulfolobales*.

Here we show that polyP is also related to biofilm formation in archaea, affecting both attachment of cells to surfaces and motility, apparently through assembly of the archaellum (archaeal motility structure), in two model crenarchaeotes *Sa. solfataricus* and *S. acidocaldarius*.

## 2. Materials and Methods

### 2.1. Strains and Growth Conditions

All strains used in this study are cited in Supplementary Table S1.

*Sa. solfataricus* M16 and its polyP (−) strain [12], as well as *S. acidocaldarius* MW001 and its corresponding polyP (−) strain were grown as before at 75 °C by shaking at 150 rpm in Brock Medium [12], pH 3 and supplemented with 0.1% (*p/v*) N-Z amine (Sigma-Aldrich^®^, Merk KGaA, Burlington, MA, USA), 0.2% (*p/v*) glucose and 0.01 mg/mL uracil only in the case of uracil autotrophs. For overexpression of PPX (described below), 0.2% (*w/v*) d-arabinose (Sigma-Aldrich^®^, Merk KGaA, Burlington, MA, USA) was added.

### 2.2. Construction of a S. acidocaldarius Mutant Lacking PolyP

To generate recombinant *S. acidocaldarius*, the *ppx* gene (Saci_2018) was amplified from *S. acidocaldarius* MW001 genomic DNA using PCR with primers 10904 and 10905 (Table S2). The obtained product was cloned into pSVAaraFX-H6 [13] using *Sap*I (New England Biolabs., Ipswich, MA, USA), resulting in pSVA12801. The plasmid was sequenced.

Before being introduced in *S. acidocaldarius* MW001, the plasmid was methylated by transformation and purification from *E. coli* ER1821 strain. Methylated plasmids were transformed to competent *S. acidocaldarius* MW001 as described in [14] and plated on Brock first selection plates. Colonies were picked and grown in Brock medium supplemented with N-Z amine and Glucose.

The overexpression of PPX enzyme was induced for 3 h with 0.2% d-arabinose while growing in liquid Brock medium with supplements. Cells were collected by centrifugation and boiled in protein buffer (0.5 M Tris-HCl, pH 6.8, 65 mM glycerol, 10% SDS, 2% 2-mercapto ethanol, 0.025% bromophenol blue) at 100 °C for 5 min.

### 2.3. PolyP Extraction and Quantification

Cultures of *S. acidocaldarius* were grown as described in Section 2.1. PolyP from *S. acidocaldarius* cells were isolated as previously described [12]. In brief, 5 mL culture was centrifuged for 15 min at 4500× *g*. Cell pellet was resuspended in prewarmed 300 μL of 4 M guanidine isothiocyanate (GITC), 50 mM Tris-HCl pH 7.0 solution. The suspension was subjected to vortex and heated for 3 min at 95 °C. Twenty µL of each sample was saved for protein quantification using Coomassie Plus Protein Assay Reagent (PIERCE™, Thermo Fisher Scientific, Waltham, MA, USA). Thirty µL of 10% SDS was added to each sample and then heated again at 95 °C for 5 min. To bind polyP, 5 µL of silica (Glassmilk) and 300 µL of ethanol (100%) were added and mixed by vortexing followed by heating at 95 °C for 30 s. After 1 min centrifugation at 13,000× *g*, the supernatant was removed and the glassmilk pellet was resuspended in 200 µL of ice cool New Wash Buffer (5 mM Tris-HCl pH 7.5, 50 mM NaCl, 5 mM EDTA, 50% ethanol). After centrifugation, glassmilk was resuspended in 100 µL of a solution containing 50 mM Tris-HCl pH 7.0, 5 mM MgCl_2_, 5 µg/mL DNase and 5 µg/mL RNase and incubated for 30 min at 37 °C to eliminate nucleic acids. The pellet was washed twice with New Wash Buffer. Finally, a total of 100 µL of polyP-containing solution was obtained and polyP was recovered from the glassmilk after repeatedly vortexing with 50 µL of water, heating at 95 °C and centrifugation. If not measured immediately, samples were frozen and stored at −20 °C.

Quantification of total P_i_ was done with EnzChek Phosphate Assay kit (Invitrogen™, Thermo Fisher Scientific, Waltham, MA, USA) following manufacturer’s instructions and expressed as nmol of P_i_ per mg of protein. Thirty µL of polyP containing solution was subjected to acid hydrolysis with 30 µL 2 N HCl for 30 min at 95 °C to release inorganic phosphate (P_i_), followed by quantification.

### 2.4. Microtitration Plates Assays

Cells were grown to stationary phase in Brock medium (pH 3.5) supplemented with 0.1% N-Z-amine and 0.2% d-arabinose or uracil in 96 wells microtitration plates covered with gas permeable sealing membrane (Breathe-Easy, Diversified Biotech, Boston, MA, USA) at 75 °C for 2 or 3 days inside a humidity chamber to avoid evaporation. The starting OD_600_ was 0.03 for *Sa. solfataricus* and 0.01 for *S. acidocaldarius*, as previously described by Koerdt et al. [15]. After 2 or 3 days, plates were cooled down, the supernatant was placed in a new plate and the OD_600_ was determined by an Epoch luminometer (BioTek instruments, Agilent Technologies, Inc., Santa Clara, USA). Ten µL of 0.5% crystal violet solution (CV) was added to each well containing the biofilm followed by incubation for 10 min. Sessile cells were washed with Brock (pH 5) medium or water, and CV attached to the biofilm was released by using 200 µL of 30% acetic acid. The CV released was determined by measuring the OD_570_. The OD_570_/OD_600_ correlation index was used to determine biofilm formation efficiency.

### 2.5. Confocal Laser Microscopy

For Confocal Laser Microscopy (CLM), cells were grown in 35 mm petri dishes (µ-dishes; Ibidi; Martinsried) [15]. Culture medium was exchanged every 24 h, and after 3 days, the supernatant was exchanged for 2 mL Brock (pH 5) and biofilm was stained with 3.6 µL of DAPI (4′,6-diamidino-2-phenylindole) (500 µg/mL), 15 µL ConA-fluoresceine (5 mg/mL) and IB4-Alexa568 (lectin IB4 from *Griffonia simplicifolia*, Invitrogen™, Thermo Fisher Scientific, Waltham, MA, USA) was used for CLM. Staining was done for 30 min in the dark at room temperature.

Biofilms were observed under microscope ZEISS Observer 1 with 63× objective

### 2.6. Adhesion Assays

For adhesion assays, 40 mL of *Sa. solfataricus* or *S. acidocaldarius* cultures with an initial OD_600_ of 0.03 and 0.01, respectively, were grown to OD_600_ of 0.5–0.7 for 24 h at 75 °C and 150 rpm in a 100 mL Schott flask with a glass slide inside. After that period, the glass slide was removed, washed twice with Brock medium (pH 5), and cells attached were fixed with 4% formaldehyde dissolved in Brock medium (pH 5). Cells and EPS were stained with 6 µL DAPI (300 µg/mL) and 15 µL (5 mg/mL) ConA dissolved in 1 mL Brock medium (pH 5) for 30 min in the dark. After staining, slides were washed twice with Brock medium and air dried. Cells in the back part of the slide were removed with 70% ethanol. Slides were observed by using the TIRF Observer 1 from ZEISS microscope with a 100× objective. Ten pictures from different fields were taken from each of 3 biological replicates and processed with Fiji (ImageJ) [16].

### 2.7. Motility Assays

Motility was analyzed on semi-solid gelrite plates. Motility plates were made with Brock medium pH 5, 0.15% gelrite and supplemented with 0.0001% N-Z amine, 0.2% d-arabinose and uracil when needed. Cells for inoculation were grown to an OD_600_ between 0.4 and 0.6, placed as a spot on the surface of the motility plates and air dried. Plates were incubated for 4 days in the case of *S. acidocaldarius* strains, and 9 days for *Sa. solfataricus* strains inside a humidity chamber. Finally, the swimming radius/area of each spot was measured.

### 2.8. Total RNA Extraction and cDNA Synthesis

To study the expression of genes of interest, cells were grown in large 150 mm petri dishes for biofilm formation during 3 days at 75 °C and no agitation inside a humidity chamber, exchanging the medium after the first 24 h. Cells were grown in Brock medium, starting at an initial OD_600_ of 0.03. For polyP (−) strains, d-arabinose was added. For planktonic cells, cultures were grown in the same conditions but in Erlenmeyer flasks.

After 3 days, the supernatant from the biofilms was removed and cells from the biofilm were washed twice with Brock medium and scraped from the bottom with a cell scraper and 20 mL of fresh medium. Biofilm and planktonic cells (10 mg wet weight) were harvested by centrifugation (7700× *g* for 15 min). Cell pellets were washed three times with Brock’s medium and lysed as previously described [17]. RNA was extracted by using TRIzol (Invitrogen™, Thermo Fisher Scientific, Waltham, MA USA) as described by the manufacturer. Remaining DNA was eliminated by adding 40 U of TURBO DNA-free DNase (Invitrogen™, Thermo Fisher Scientific, Waltham, MA, USA) following manufacturer’s instructions. 0.8 µg of total RNA was reverse transcribed for cDNA synthesis using ImProm-II (Promega, Madison, WI, USA), 0.5 µg of random hexamers (Promega, Madison, WI, USA) and 3 mM MgCl_2_ for 1 h at 42 °C. Three biological replicates were used for every experimental condition.

### 2.9. Primer Design and Real-Time RT-PCR

Primers for qRT-PCR were designed using Primer3 Software and the annotated genome of *Sa. solfataricus* P2 *and S. acidocaldarius* DSM639, as they are the WT strains [14,18] (Table S1). All primers are listed in Table S2. To check primer specific annealing and optimal melting temperature, PCR reactions were carried out with Taq DNA polymerase from Promega following manufacturer’s instructions and the products were separated by gel electrophoresis (1% agarose).

Gene expression was analyzed with the 96-well PikoReal-Time PCR System (Thermo Fisher Scientific, Waltham, USA). Five µL of KAPA SYBR^®^ FAST 2X (Sigma-Aldrich^®^, Merk KGaA, Burlington, USA) was used along 0.2 µL of each primer and 0.5 µL of a 1:20 dilution of the cDNA.

The efficiency of each pair of primers was calculated from the average slope of a linear regression curve, constructed from qPCRs using a 10-fold dilution series (10 pg–10 ng) of *Sa. solfataricus* M16 or *S. acidocaldarius* MW001 chromosomal DNA as template. Cq values (quantification cycle) after 40 cycles were automatically determined by PikoReal Software 2.1 (Thermo Fisher Scientific, Waltham, MA, USA). Cq values of each transcript of interest was standardized to the Cq value of the *16s* and/or *30s* rRNA gene. At least 3 biological replicates of each assessed condition and 2 technical replicates per qPCR reaction were performed. *Rps2P* and *16S* rRNA were used as housekeeping genes.

### 2.10. Starvation Induction Experiment

*S. acidocaldarius* MW001 and polyP (−) were grown in Brock medium and supplemented with 0.1% (*p/v*) N-Z amine (Sigma-Aldrich^®^, Merk KGaA, Burlington, USA), 0.2% (*p/v*) dextrose and 0.01 mg/mL uracil only in the case of *S. acidocaldarius* MW001. For overexpression of PPX, 0.2% (*w/v*) d-arabinose (Sigma-Aldrich^®^, Merk KGaA, Burlington, USA) was added. A control of *S. acidocaldarius* MW001 with plasmid pSVA12801 without induction was used.

After reaching an OD600 of 0.4–0.5, cells were centrifuged for 10 min at 4400 rpm and 70 °C and resuspended in starvation medium without supplements, but with uracil and d-arabinose when needed. Cells were grown for 4 h at 75 °C and 150 rpm, and then used for Transmission Electron Microscopy.

### 2.11. Transmission Electron Microscopy

Five µL of cells from the starvation experiment were applied on a glow-discharged 300 mesh formvar and carbon-coated copper grid (Plano GmbH, Wetzlar, Germany) and incubated for 10 s. The excess liquid was blotted away and the application step was repeated 8 times subsequently. Afterwards cells were stained with 2% (*w/v*) uranyl acetate. Imaging was performed using Zeiss Leo 912 Omega (Tungsten) (Carl Zeiss, Oberkochen, Germany) operated at 80 kV. Images were taken with Dual Speed 2K-On-Axis charged-coupled device (CCD) camera TRS, Sharp-Eye (TRS Systems, Moorenweis, Germany).

### 2.12. Statistical Analysis

Data obtained was subjected to analysis of variance (ANOVA) and a Bonferroni’s test in the Prism 8 GraphPad software.

## 3. Results

### 3.1. Biofilm Formation

Microtiter plates assays were performed to compare biofilm formation of background and polyP (−) strains of both *Sa. solfataricus* and *S. acidocaldarius*. Lack of polyP in *S. acidocaldarius* MW001 transformed with pSVA12801 was confirmed after 3 h post induction of *ppx* with 0.2% d-Arabinose (Figure S1), as described before for *Sa. solfataricus* [12]. A control of cells with plasmid but no induction was use for some experiments and they will be referred as *Sa. solfataricus* M16-PPX and *S. acidocaldarius* MW001-PPX.

**Figure 1:**
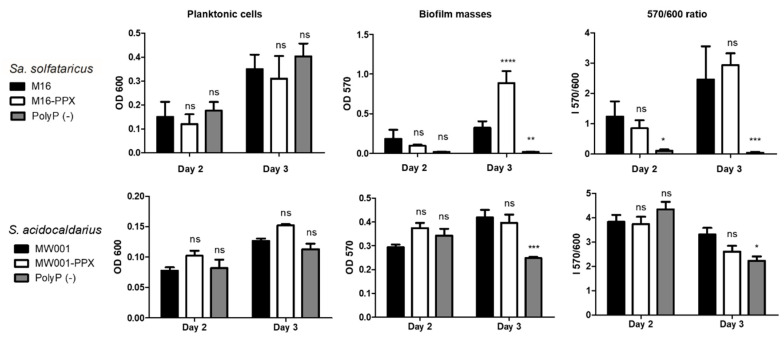
Effect of the lack of polyphosphates (polyP) in biofilm formation in *Sa. solfataricus* and *S. acidocaldarius* on days 2 and 3 of growth. Microtitrate assay of background strains (M16 and MW001), cells transformed with plasmid but no induction of *ppx* (M16-PPX and MW001-PPX) and polyP (−) cells from each species. The biofilm mass corresponds to Cristal violet attached to the biofilm measured at 570 nm. ANOVA test: **** indicating *p* ≤ 0.0001, *** *p* ≤ 0.001, ** *p* ≤ 0.01, * *p* ≤ 0.05 and ns: no significative.

*Sa. solfataricus* biofilms of polyP (−) and background strains at days 2 and 3 of growth showed significant differences as indicated by the 570/600 nm index (Figure 1, 570/600 ratio panel). The number of planktonic cells remained similar for all conditions (Figure 1, upper panels, Planktonic cells) as was previously described [12]. The differences were therefore mainly due to changes in biofilm formation as shown by crystal violet staining (biofilm) (Figure 1, Biofilm masses).

A similar but less pronounced effect could be observed for *S. acidocaldarius* (Figure 1, lower panels), with polyP (−) strain showing decreased levels of biofilm formation when compared to the background strain after 3 days of growth. Planktonic cells showed no significant differences. In addition, growth curves of both strains were similar (Figure S2). In both cases, cells with plasmid but no induction of ppx (M16-PPX and MW001-PPX) showed similar biofilm production compared with their background counterparts.

Here we have therefore shown that comparable to what is known from many bacteria [5,6,7], *Sulfolobales* polyP (−) strains also showed reduced biofilm formation.

### 3.2. Confocal Laser Microscopy

**Figure 2:**
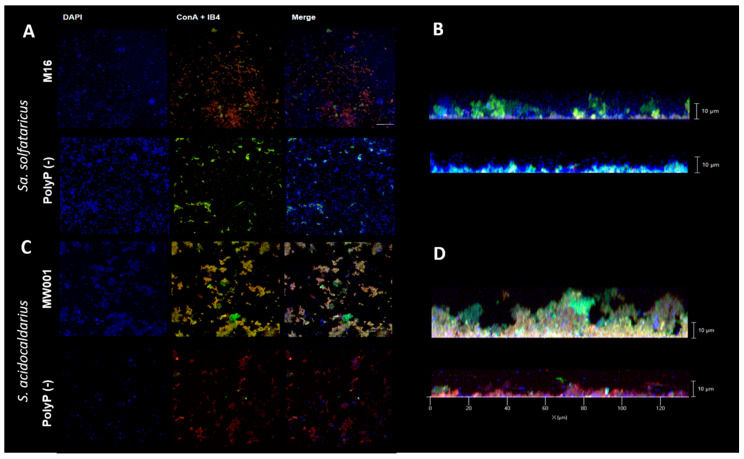
Confocal Laser Microscopy showing differences between wild type (WT) and polyP (−) strains biofilms in day 3. Biofilms were stained with DAPI (4′,6-diamidino-2-phenylindole), ConA and IB4. Biofilms from (**A**) M16 and polyP (−) strains of *Sa. solfataricus* and (**C**) MW001 and polyP (−) of *S. acidocaldarius* in DAPI channel (blue), merge of ConA (green) and IB4 (red) channels (extracellular polymeric substances (EPS)) and merge of all three channels. (**B**,**D**) Z stack of WT and polyP (−) strains in both species, merge with all three channels.

To characterize the biofilm morphology of polyP (−) strains, CLM was done on 3 days old biofilms stained with DAPI (DNA), ConA (staining α-mannopyranosyl and α-glucopyranosyl residues) and IB4 (for α-d-galactosyl residues). The biofilm of *Sa. solfataricus* M16 showed a morphology similar to that previously described for *Sa. solfataricus* M16 strain (Figure 2) [15], with some cumulus of EPS and a uniform distribution of cells, but the biofilm was thinner in our strain (Figure 2B). As previously described, *Sa. solfataricus* M16 excretes EPS in low amounts [15] (Figure 2A, ConA+IB4 channels).

*Sa. solfataricus* polyP (−) biofilms were thinner than the background strain biofilms (6 versus 10 µm) and contained less cells and very low amounts of EPS (Figure 2A). Additionally, the composition of the glycans present in the biofilm seemed to be different when comparing both strains: biofilms of *Sa. solfataricus* polyP (−) mainly contained α-mannopyranosyl and α-glucopyranosyl residues, (Figure 2A, green, ConA), whereas its background strain contained more α-d-galactosyl residues (Figure 2A, red, IB4).

*S. acidocaldarius* polyP (−) also showed a thinner biofilm when compared to the background strain (Figure 2D). Moreover, lower amounts of α-mannopyranosyl and α-glucopyranosyl sugars could be observed (Figure 2C, green, ConA).

### 3.3. Phenotypes Related to Surface Structures: Adhesion to Glass Surface and Motility Assays

The archaellum in *Sa. solfataricus* and *S. acidocaldarius* plays an important role in attachment to surfaces [19,20]. In *S. acidocaldarius* there is an additional surface appendix that is involved in adhesion and biofilm initiation, called Adhesive Archaeal Pilus (Aap-pilus) [21].

**Figure 3:**
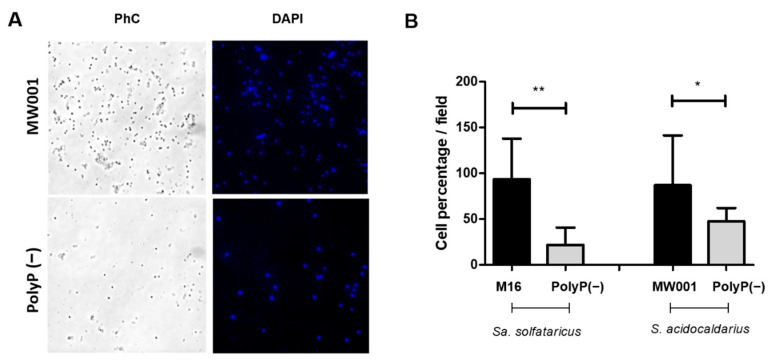
Effect of the lack of polyP in *Sa. solfataricus* and *S. acidocaldarius* cells attachment to a glass surface after 24 h. (**A**) Microscopical image of MW001 and polyP (−) strains from *S. acidocaldarius* attached to glass slides. Cells were fixed with formaldehyde and stained with DAPI. Phase contrast (PhC) and DAPI channels are shown. (**B**) Mean of the percentage of cell numbers per field. Each value was calculated in function of the average in the number of cells per field in the respective background strain (*Sa. solfataricus* M16 or *S. acidocaldarius* MW001). ANOVA test: * *p* ≤ 0.05 and ** *p* ≤ 0.01.

To test if the surface attachment of polyP (−) strains was also impaired, all strains were grown in shaking cultures each with a glass slide inside. Both polyP (−) strains showed reduced attachment to the glass surfaces when compared to their corresponding background strains (Figure 3). This difference in initial cell attachment could (partially) explain the observed reduced biofilm formation of the polyP (−) strains (Figure 1).

**Figure 4:**
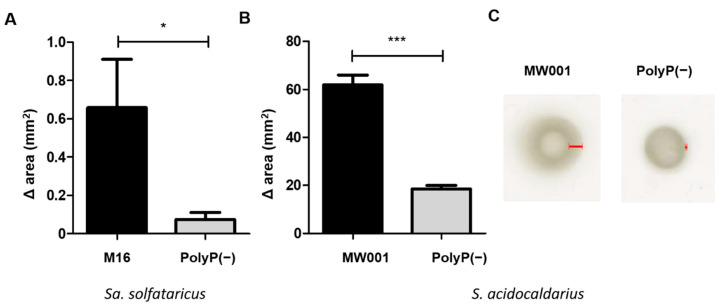
Motility assays of polyP (−) and WT strains of *Sulfolobales*. (**A**) *Sa. solfataricus* M16 and (**B**,**C**) *S. acidocaldarius* MW001. The results represent an average of at least 10 spots. ANOVA test: * *p* ≤ 0.05 and *** *p* ≤ 0.001.

To further study other characteristics previously described for polyP (−) mutants in bacteria [7,8], motility assays in semi solid gelrite plates were done. PolyP (−) strains had only 30% swarming motility compared to the respective background strains (Figure 4). The observed phenomena—adhesion, motility, and biofilm formation—are all related to the mentioned surface structures in the cells, such as archaellum and adhesive pili [19,20]. Thus, polyP might be affecting levels, and/or the function of these structures.

### 3.4. Surface Structures Levels and Archaellum Assembly in PolyP (−) Mutants

**Figure 5:**
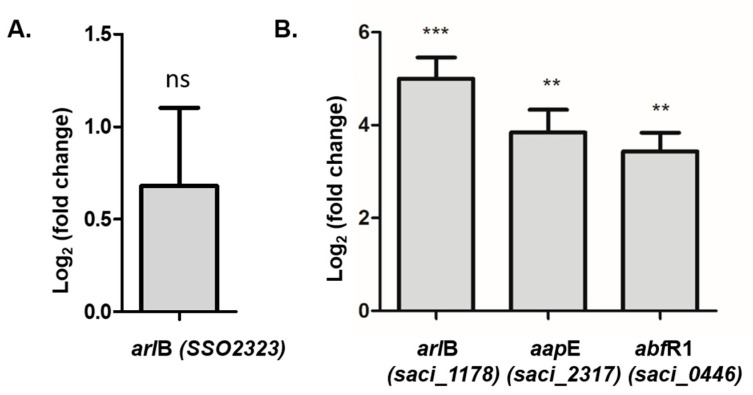
Relative gene expression of *arl*B, *aapE* and *abfR1* genes as measured by qRTPCR in polyP (−) versus WT in planktonic cells. (**A**) *Sa. solfataricus* (**B**) *S. acidocaldarius*. Wilcoxon Signed Rank Tests where *** *p* ≤ 0.001, ** *p* ≤ 0.01 and ns: no significant.

To find out whether the observed reduced motility of the polyP (−) strains was due to an altered expression of the components of the archaellum (the archaeal motility structure), the transcript levels of *arlB,* encoding the structural component of the archaellum, were measured by qRT-PCR and compared between polyP (−) and background strains. In *Sa. solfataricus,* levels of *arl*B (SSO2323) did not show a significant difference (Figure 5A), whereas there was a higher *arl*B mRNA level in the polyP (−) strain of *S. acidocaldarius* (Saci_1178; Figure 5B). These results do not explain the reduced adhesion of the *Sa. solfataricus* polyP (−), nor the reduced motility in both polyP (−) strains (Figure 4A).

The Aap-pilus is also involved in initial cell adhesion of *S. acidocaldarius* [19]. Thus, the levels of *aapE* mRNA, encoding for the ATPase energizing the assembly of this pilus were determined (Figure 5B). Almost 4 times higher levels of this component were found in polyP (−) cells compared to MW001.

Finally, the levels of *abf*R1 (Saci_0446) were measured. This gene codes for a protein that acts as a negative regulator of biofilm formation [10]. When phosphorylated AbfR1 does not inhibit biofilm formation and decreases its own expression [11], therefore it was of interest to test if the lack of polyP might affect the expression of this gene. As seen in Figure 5B, the expression levels of this gene were upregulated in the polyP (−) strain. This result could explain both the lower amount of biofilm formed by the polyP (−) strain in *S. acidocaldarius*, as well as the lower amounts of EPS seen by microscopy (Figure 2C,D).

### 3.5. Archaellum Assembly in PolyP (−) Mutants

It is not known how lack of polyP affects motility in cells, but it is possible that it is related to its role as an energy reservoir [4].

The results obtained here showed higher levels of the archaellin gene was being expressed in *S. acidocaldarius* polyP (−) strain. Besides the role of ATP in the archaellum rotation it is also known to play a function in the archaellum assembly [22]. Therefore, to determine whether the archaellum and other structures were being properly assembled on the cell surface, transmission electron microscopy (TEM) of *S. acidocaldarius* MW001 and polyP (−) cells was done.

**Figure 6:**
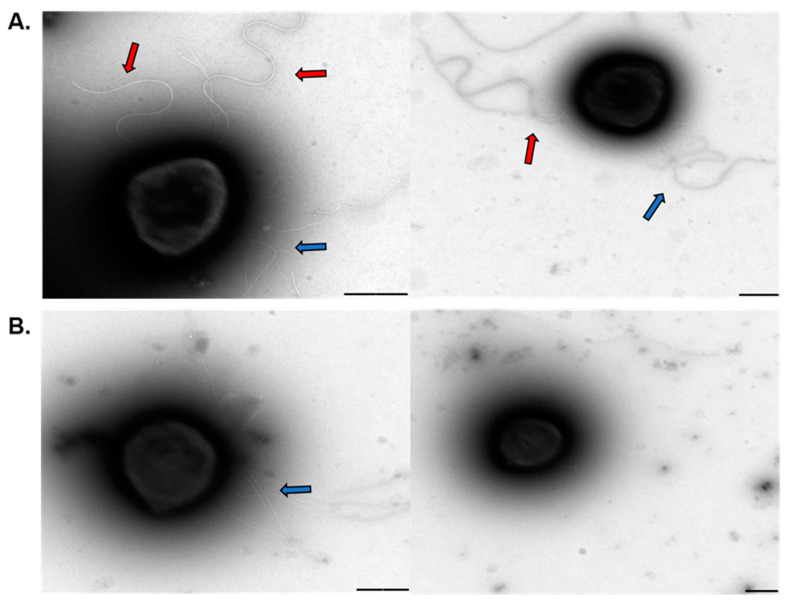
Transmission Electron Microscopy of *S. acidocaldarius* cells after starving conditions for archaellum induction. (**A**) MW001 (**B**) PolyP (−). Red arrows point to archaellum filaments. Blue arrows point to pili. The scale bars are 500 nm.

Archaellum formation can be induced by starvation [23]. *S. acidocaldarius* MW001 and polyP (−) strain subjected to starvation showed archaella as seen in *S. acidocaldarius* wild type strain (Figure 6A, red arrows) and in the control strain of *S. acidocaldarius* MW001-PPX (plasmid without induction) (Figure S3). In the polyP (−) strain, no archaellated cells could be perceived (Figure 6B). Pili could be seen in both strains, but in higher numbers in the WT strain (blue arrows).

This lack of archaellation in cells could explain the reduced biofilm formation and motility deficiency on semi solid gelrite plates (Figure 1 and Figure 4).

## 4. Discussion

Overexpression of the *ppx* gene in both *Sa. solfataricus* and *S. acidocaldarius* eliminates polyP from their cells. Therefore, the use of these overexpression strains allowed to study the effect of polyP on biofilm formation, adhesion, and motility in archaeal cells.

Similar to what was observed for cells lacking the archaellum [19,21], polyP (−) cells exhibited a reduced surface-attachment and were not motile. Moreover, the *S. acidocaldarius* polyP (−) strain produced a compact biofilm, with less amounts of EPS as observed for the Aap-pilus deletion mutant and the double deletion mutant for archaellum and Aap-pilus [21]. The results presented here therefore suggest that similar to what is known in bacteria, polyP in *Sulfolobales* functions in biofilm formation and motility by regulating the production of cell-surface structures that are known to be involved in the initial cellular swimming and attachment to surfaces [19,20,21].

The exact mechanism of how polyP regulates biofilm formation is not known. In *E. coli*, the degradation of the polymer at the beginning of stationary phase is related to LuxS and the formation of biofilm [9]. In *Sulfolobales*, no Quorum sensing system is known. Therefore, polyP must regulate biofilm formation in a different way.

**Figure 7:**
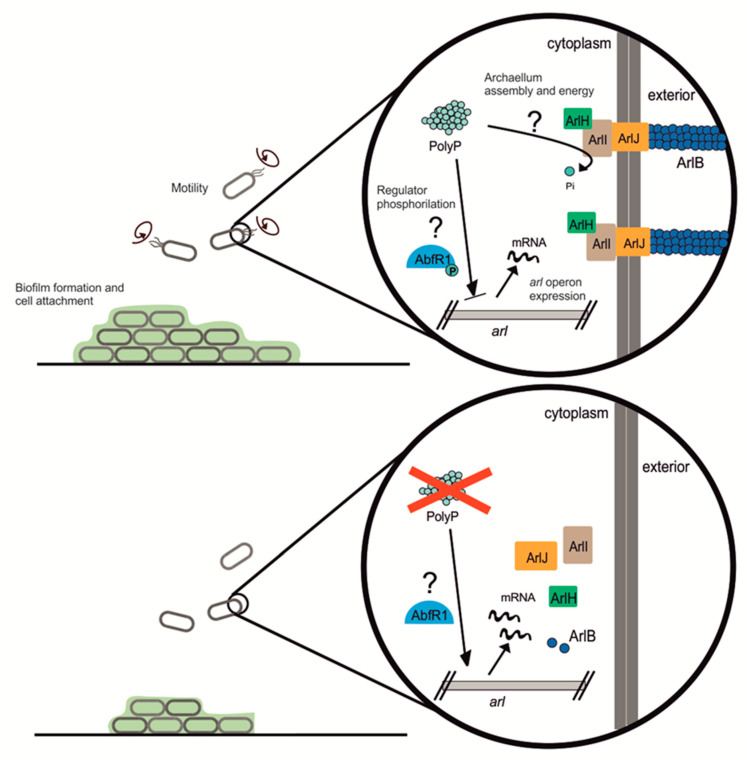
Cartoon showing the possible role of polyP in motility, adhesion, and biofilm formation in *Sulfolobales*.

*S. acidocaldarius* contains two main regulators from the Lrs-14 family: Saci_1223 and AbfR1 [10]. AbfR1 was of particular interest since it is itself regulated by phosphorylation. AbfR1 acts by inhibiting biofilm formation via induction of the *arl* operon and reduction of EPS synthesis [11] (see Figure 7). AbfR1 also induces its own expression. However, when phosphorylated, AbfR1 cannot bind its regulation targets (*arl*B promoter and its own *abf*R1 promoter regions). Here we found that *abf*R1 mRNA levels are higher in the polyP (−) strain, which explains in part the lower amount of biofilm formation. A possible explanation to this phenomenon is that the lack of polyP affects the regulation of AbfR1 by impairing phosphorylation, which would lead not only to the over-expression of AbfR1 itself but also the overexpression of the *arl* operon (Figure 7).

Deficient protein phosphorylation-disturbing expression of the archaellum genes was seen before for *S. acidocaldarius* [24], where cells lacking protein phosphatases genes (Saci_0545 and Saci_0884) showed overexpression of archaellum but less motility on semi-solid plates compared with the WT counterpart. However, *Sa. solfataricus* AbfR1 (SSO_0458) does not harbor the amino acids proposed to be phosphorylated in *S. acidocaldarius* (Y84 and S87) [11]. It is therefore unclear how polyP is affecting phosphorylation of this regulator in both species. It is possible that polyP might also function in providing energy for assembly of the archaellum and Aap-pili [22]. The assembly of the archaellum is energized by ATP hydrolysis [25]. In *E. coli ppk* and *ppx* knockout mutants had lower expression of genes coding for fimbria and flagellum and were not flagellated as seen under the microscope [26]. Although *Pseudomonas sp.* B4 polyP (−) mutant showed lower levels of flagellin, intact flagella were also seen at its surface [8].

It is interesting to consider that *ppx*-gene overexpression could generate an artificial ATP-consuming futile cycle in the cells, which is expected to lower the availability of ATP and thus the general energy load of the microorganisms. In future studies, it will be of importance to consider this possible effect, especially since deprivation of ATP might also affect archaella assembly and its movements. In addition, measuring the effects on cellular ATP concentration or heat generation (by microcalorimetrically), or on the relative fitness of the cells, would also be of interest in potential studies.

It is possible that an eventual lack of energy might take place in both *Sa. solfataricus* and *S. acidocaldarius* strains that do not accumulate polyP due to the overexpression of their respective PPX enzymes. This might create a futile cycle due to the constant polyP degradation, which in turn can cause an eventual lack of energy in these Crenarchaeotes. This situation might in turn explain the lack of motility and low biofilm formation phenotypes reported in this work. It is known that both of these strains normally accumulate low levels of polyP in their cytoplasm [27]. Consequently, the amount of ATP required for the polymer synthesis should not be high. A bacterial kind of PPK type has not yet been identified in *Sulfolobales*. In addition, *Sulfolobus* species possess an ATP synthase enzymatic complex able to use the proton motive force to regenerate ATP [28]. Considering these species grow at acid pH, ATP production could be favored in a natural way under their growth conditions. Each phosphate extension of the polyP polymer spends one ATP molecule and generates one of ADP. On the other hand, polyP degradation by the PPX enzyme would release one molecule of inorganic phosphate by each broken phosphodiester bond. Given these facts, one can consider that these archaeal strains could use both ADP and Pi substrates together with the energy of proton motive force to regenerate ATP from both polyP synthesis and degradation.

## 5. Conclusions

Even though polyP plays many functions in both eukaryotic and prokaryotic species, only little is known about the role of polyP in archaea. Here we report that polyP is important in biofilm formation, adhesion, motility, expression, and assembly of the archaellum in crenarchaeotes *Sa. solfataricus* and *S. acidocaldarius*. polyP (−) cells were not archaellated and showed less pili compared to the background strains used in this study. The lack of archaellum and pili in cell surface explains the lower motility and adhesion to glass surface, as well as some of the characteristics of the biofilm structure in these strains. We propose polyP might be affecting phosphorylation of proteins such as AbfR1, which is involved in biofilm formation as well as expression of Arl operon. At the same time, lack of polyP affects the assembly of the surface structures. Other levels of regulation should not be discarded meanwhile this study opens new questions about different roles of polyP in archaea.

This is the first evidence that polyP influences the mentioned phenomena in Archaea. By using genetically tractable archaea such as *S. acidocaldarius*, future studies would help us to determine the exact mode of regulation of archaeal polyP.

## Data Availability

The data presented in this study are openly available.

## Supplementary Materials

The following are available online at https://www.mdpi.com/2076-2607/9/1/193/s1, Figure S1: Lack of PolyP in *S. acidocaldarius* PolyP (−) strain, Figure S2: Growth curves comparing *S. acidocaldarius* MW001 and PolyP (−) strains, Figure S3: Representative TEM image from *S. acidocaldarius* MW001-PPX, Table S1: Strains used in this work, Table S2: Primers used in this work.
