# Supplementary material for: The Role of Polyphosphate in Motility, Adhesion, and Biofilm Formation in Sulfolobales
Source: Microorganisms. 2021 Jan 18;9(1):193. doi: 10.3390/microorganisms9010193 (PMC7831078; doi:10.3390/microorganisms9010193)
Supplement: Supplementary file 1 [file microorganisms-09-00193-s001.pdf]

SUPPLEMENTARY DATA

SUPPLEMENTARY FIGURES

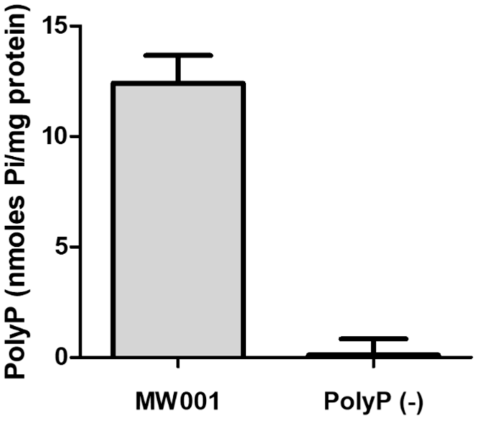

Figure S1

Lack of PolyP in *S. acidocaldarius* PolyP (-) strain. Both *S. acidocaldarius* MW001 and PolyP (-) were induced with D-arabinose during the exponential growth phase to overexpress *ppx* in the later. PolyP levels were measured after 4 h postinduction.

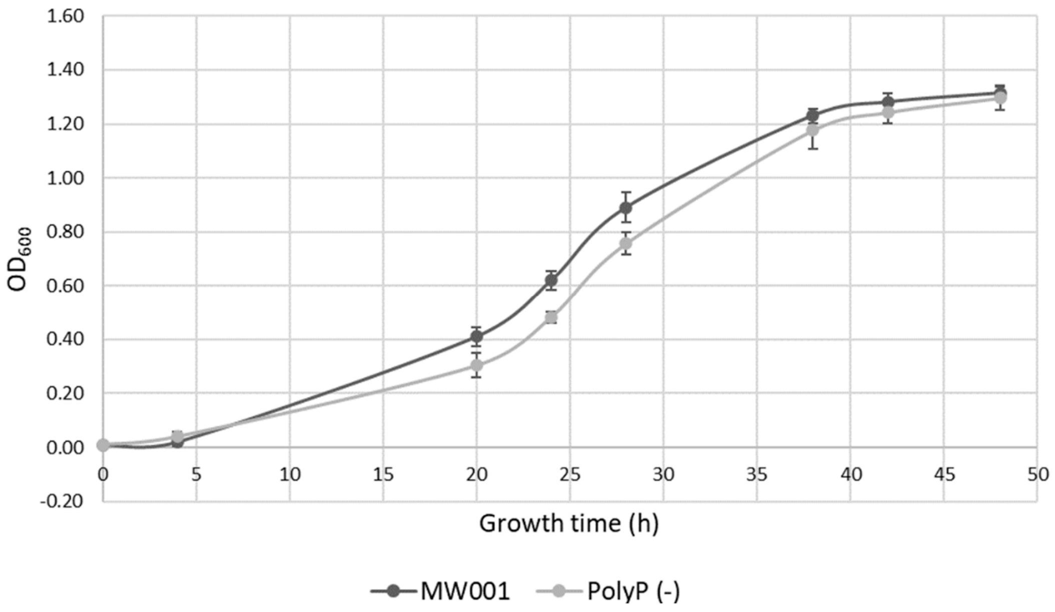

Figure S2

Growth curves comparing *S. acidocaldarius* MW001 and PolyP (-) strains. Cells were grown at 75°C and 150 rpm in Brock medium supplemented as described, with 0.2% D-arabinose and additionally uracil for MW001. A pre culture for PPX induction was used in order to have cells lacking PolyP from the start of the experiment.

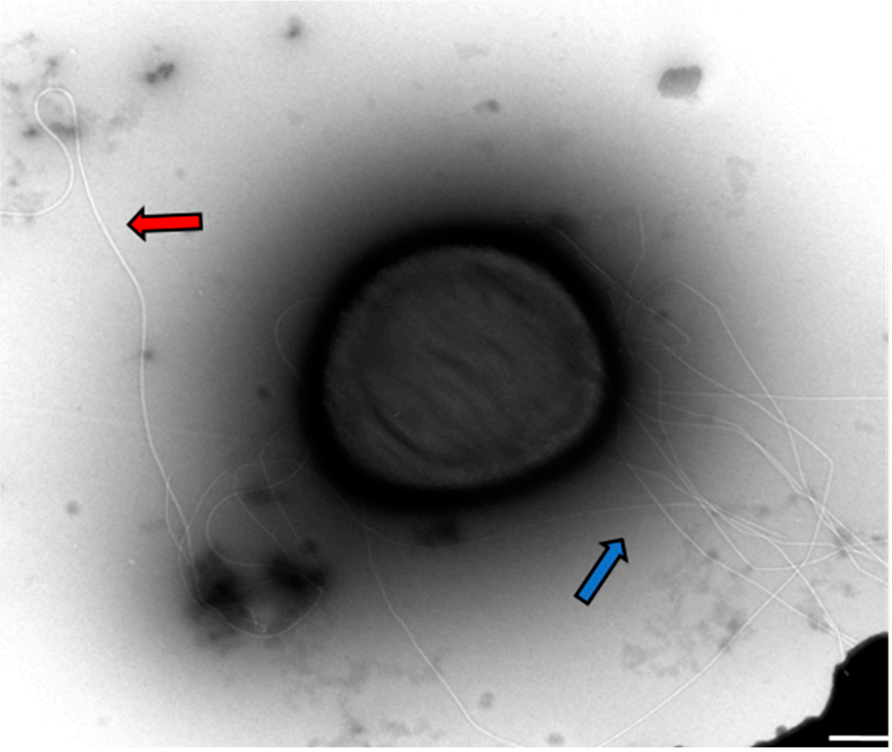

Figure S3

Representative TEM image from *S. acidocaldarius* MW001-PPX. When *ppx* is not induced, cells still show archaellum (red arrow) and pili (blue arrow) even in the plasmid presence. White bar represents 250 nm.

SUPPLEMENTARY TABLES

TABLE S1.

Strains used in this work

Strain	Genotype / Phenotype	Reference
Sa. solfataricus		
**M16**	Uracil-auxotrophic mutant derived from *Sa. solfataricus* P1 (DMS1616)	[18]
**PolyP (-)**	Contains plasmid J0503-r_ppxS.so over expressing *Sa. solfataricus* PPX in presence of D-arabinose and complemented for *pyr*EF. Does not accumulate PolyP.	[12]
S. acidocaldarius		
**MW001**	Deletion of *pyrEF* (91-412 bp) in *S. acidocaldarius* DSM639. Uracil-auxotrophic mutant	[14]
**PolyP (-)**	Contains plasmid pSVA12801 over expressing *S. acidocaldarius* PPX in presence of D-arabinose and complemented for *pyr*EF. Does not accumulate PolyP.	This study

TABLE S2.

Primers used in this work

Cloning

Name	Sequence (5´ 3')	Description
**10904**	ATATATGCTCTTCTAGTCGATATGCGGTAATAGACACGGGTTAC	Forward primer for cloning *saci_2018* into pSVAaraFX-H6 with *Sap*I restriction site
**10905**	TATATAGCTCTTCATGCTAGCACACCAGCCACTGACGACTCTAC	Reverse primer for cloning *saci_2018* into pSVA araFX-H6 with *Sap*I restriction site

qPCR

Gen	Protein	Primer Foward (5’ → 3´)	Primer Reverse (5’ → 3´)
**16s SSO**		GGTAGCAAGTAGACCCTATGC	GGTATCAGCAAAAGCGACAA
**SSO0067**	30s Rps2p	GGTAGCAAGTAGACCCTATGC	GGTATCAGCAAAAGCGACAA
**SSO2323**	Archaellin	TGCAGTAAACGAGATGGCTG	TGACACAGAAAGCCAAATCCAC
	16s Saci	CTTTCGAGGTAGCATCCCG	GTGAAATGTCCGGTTAAGTCC
**Saci_1178**	Archaellin	ACTGCGTCTACTGCGTTATCTTTATC	GGAGATAAGTCTACACTAGATACACCA
**Saci_2317**	AapE	GGGGCTCATTCATTAACCTCTT TAT	GAAAGGAGATTACGCTAATGGA CTT
**Saci_0446**	AbfR1	TCACCCTTTCCAACCCTATC	GCTAGTACCTACACCGAGAC
